# Prophylactic effect of herbal-marine compound (HESA-A) on influenza A virus infectivity

**DOI:** 10.1186/1472-6882-14-131

**Published:** 2014-04-07

**Authors:** Parvaneh Mehrbod, Aini Ideris, Abdul Rahman Omar, Mohd Hair-Bejo

**Affiliations:** 1 Faculty of Veterinary Medicine, Universiti Putra Malaysia, 43400 Serdang Selangor, Malaysia; 2 Institute of Bioscience, Universiti Putra Malaysia, 43400 Serdang, Selangor, Malaysia

**Keywords:** HESA-A, H1N1, Influenza virus, HA, MTT

## Abstract

**Background:**

Influenza virus is still a severe respiratory disease affecting human and other species. As conventional drugs are not recommended for long time because of side effects and drug resistance occurrence, traditional medication has been focused as alternative remedy. HESA-A is a natural compound from herbal-marine origin. Previous studies have reported the therapeutic properties of HESA-A on psoriasis vulgaris and different types of cancers and we also showed its anti-inflammatory effects against influenza A infection.

**Methods:**

This study was designed to investigate the potential properties of HESA-A as prophylaxis or treatment. To investigate the prophylaxis or treatment activities of HESA-A, Madin-Darby Canine Kidney (MDCK) cells were exposed to HESA-A and influenza A virus in different manners of exposure and different time intervals. The results were evaluated by MTT and HA assays.

**Results:**

It was found that HESA-A is much more effective against influenza cytopathic effects when it is applied for prophylaxis and also in concurrent treatment (p ≤ 0.05) but not in post-infection treatment (p ≥ 0.05).

**Conclusion:**

In conclusion, HESA-A is significantly effective against influenza replication in prophylaxis application affecting the virus penetration/adsorption to the cell without any toxic effect on the cell viability.

## Background

Influenza A virus as a member of the *Orthomyxoviridea* family results in significant morbidity and high rates of mortality worldwide annually. Vaccines which are the best option to prevent this infection cannot be trusted because of the lag time between virus identification and vaccine distribution which may cause devastating outcomes. Conventional approved antiviral drugs such as amantadine and oseltamivir [1] which are helpful to control the spread of influenza disease from the host cell [2] may not be suggested any longer due to various genetic drift and shift mutations which cause alterations in the antibody-targeted surface glycoproteins [3] and negative side effects and rapid emergence of resistant variants [4,5]. Therefore, they are not recommended for uncontrolled usage [6]. Thus, additional antiviral alternatives preferably with natural origin easily available for the prevention/treatment of influenza A infections with low/no side effect is highly demanded.

HESA-A is an active natural biological compound from herbal-marine origin, with a general composition of organic, inorganic and aqueous fractions [7]. Previous studies have reported the therapeutic properties of HESA-A against psoriasis vulgaris, breast cancer and choroidal metastasis [8-10]. In our previous study on HESA-A, we showed the antiviral activity of this compound on influenza replication by affecting hypercytokinemia decreasing TNF-α and IL-6 expression levels [11]. In the current study we evaluated the preventive or treatment activity of HESA-A on influenza replication in different exposures and time intervals.

## Methods

### Reagents and chemicals

Cell culture media and penicillin-streptomycin solution 100× were purchased from Mediatech Cellgro Company (Northbrook, Illinois, USA). Fetal bovine serum (FBS) was purchased from PAA Laboratories (Pasching, Austria). HESA-A was kindly provided by Dr. Amrollah Ahmadi, Tehran University of Medical Sciences, Tehran, IRAN. (3-(4, 5-dimethylthiazol-2-yl)-2, 5-diphenyltetrazolium bromide, MTT), and Tosylamide Phenylethyl Chloromethyl Keton-treated Trypsin (Trypsin TPCK) were purchased from Sigma (Saint Louis, Missouri, USA).

### HESA-A preparation

Briefly, it was dissolved in normal saline, shaken for 30 minutes and filtered to become homogenate. Prior to its use, the stock solution (0.8 mg/ml, pH 7.4) was sterilized through 0.22 μm syringe filter [12]. It was diluted with DMEM to 0.025 mg/ml concentration as EC_50_[11].

### Cell culture and influenza A virus propagation

Influenza A virus strain New Jersey/8/76; H1N1 [A/NJ (H1N1)] was purchased from American Type Culture Collection (ATCC) (reference number: VR-897™) which was propagated on Madin Darby canine kidney (MDCK) cell line (CCL-34™). Cells were grown in Dulbecco's modified Eagle’s medium (DMEM) containing 10% heat-inactivated FBS, 100 units/ml penicillin G and 100 μg/ml streptomycin. Cultured cells were incubated at 37°C in the presence of 5% CO_2_. To propagate influenza A virus, cells were washed with PBS to remove residual FBS and were infected with influenza A virus at MOI of 0.5 in serum-free DMEM for 60 min at 37°C (adsorption phase). Progeny virus was harvested three days post-infection. Standard hemagglutination test (HA) was carried out to measure the viral titer [11].

### HESA-A anti-viral effect on the virus

MDCK cells seeded in 96-well plate were exposed to different combination treatments of HESA-A and influenza A virus (100 TCID_50_/0.1 ml) in different time intervals. HESA-A in a non-cytotoxic concentration was used for antiviral assays. It was added to MDCK cells before, concomitantly with or after H1N1 infection in 1, 8 and 24 hr time points. In brief, monolayers of MDCK cells were treated with HESA-A for 1, 8 or 24 hr, which was washed away before infection with H1N1 for 1 hr (pre-treatment assay), HESA-A and H1N1 were added to the cell layer together during the 1, 8 or 24 hr infection period (co-treatment assay), or HESA-A was added for 1, 8 or 24 hr right after the infection period for 1 hr (post-treatment assay). After all the treatments in allocated time, cells were washed with PBS and covered with medium containing Trypsin_TPCK (1 μg/ml) for 48 hr.

### MTT cell viability assay

MDCK cells were incubated in 96-well microplate (Nunc, Denmark) for 24 hr at 37°C to reach the confluency. Following treatments based on the experimental design, colorimetric MTT assay was performed according to Mehrbod et al. [13]. Briefly, the media was removed and 100 μl of 1× MTT (3-(4, 5-dimethylthiazol-2-yl)-2, 5-diphenyltetrazolium bromide, Sigma) was added to each well. Following incubation at 37°C for 2–3 hr, 100 μl of DMSO was added and mixed thoroughly to release the colour. The absorbance of colour in the solution was analyzed at 540 nm with microplate reader machine (BioTek EL 800, US) to calculate viability of the cells by one way ANOVA, SPSS.

### Hemagglutination assay

To evaluate the presence of the virus in cell culture, either virus-inoculated or combined-treated samples, supernatants of the culture media were exposed to chicken red blood cells (cRBCs) (0.5%). The assay was carried out as described previously by Mehrbod et al. [13]. The significant differences were analyzed using one way ANOVA, SPSS.

### Statistical analysis

The data expressed as mean ± SD was gathered and analyzed using analysis of variance (ANOVA) Tukey post-hoc test (SPSS 18.0). Sample values between different groups and treatments with p ≤ 0.05 were considered statistically significant.

## Results and discussion

**Figure 1:**
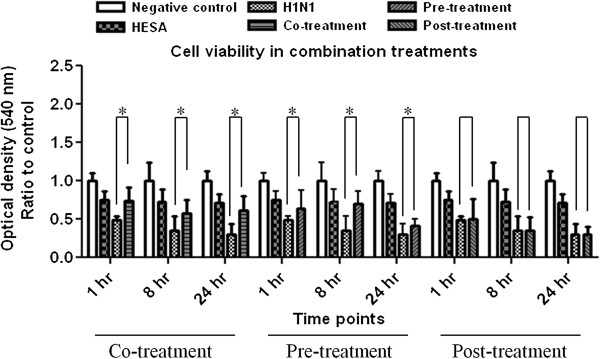
**Cell viability in combination treatments.** Data shows the MTT assay results for cell viability of HESA-A treatments against influenza A virus H1N1 CPE in different exposure types and time points. Data are presented as mean ± SD. *: Significantly different from values obtained for co- & pre-treatments as compared to the virus sample (p < 0.05).

In this experiment, to evaluate the protective effect of non-toxic concentration of HESA-A (0.025 mg/ml) in combination treatments with H1N1 in different time points, MTT viability test was conducted following incubation periods. As expected, HESA-A treatment in 0.025 mg/ml concentration did not show any significant difference with the control (p ≥ 0.05), while H1N1 treatments caused significant decrements in cell viability as compared to the control (p ≤ 0.05). However, there was significant increment in optical densities (ODs) in different combination exposures as compared to virus-inoculated sample (p ≤ 0.05) except for post-treatments (p ≥ 0.05). The results are shown in Figure 1.

**Figure 2:**
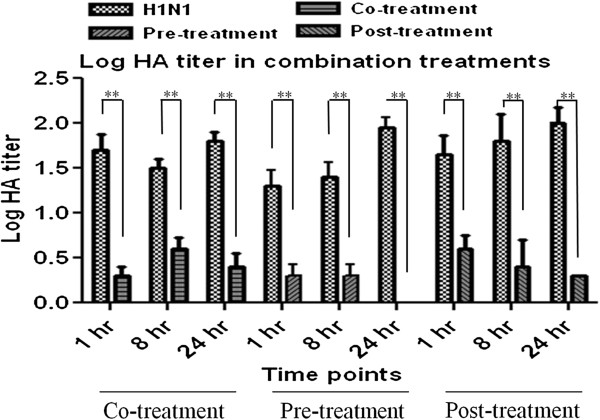
**Log HA titer reduction in HESA**-**A treatments against H1N1.** The HA data showed highly significant decrements in all combination treatments as compared to the H1N1 sample. Data are presented as mean ± SD. **: Highly significantly different from values obtained for combination treatments as compared to the virus sample (p < 0.01).

Based on hemagglutination titration, the inhibitory effect of HESA-A on viral activity was shown by highly significant reduction in Log HA titre in all combination treatments (p < 0.01). The results are shown in Figure 2.

Influenza A virus is the major cause of respiratory tract infections in a variety of species [14]. Unsuccessful treatment of this disease is related to its constant evolving genome which makes it extremely difficult to develop effective vaccines as well as antiviral drugs [15]. Thus, developing novel anti-influenza compounds, preferably of natural origin and low side effects are required [16,17]. HESA-A which is a patented natural product in IRAN, has been used against different types of diseases like wide range of cancers, psoriasis vulgaris and autoimmune diseases by decreasing the inflammatory responses and improving the patients’ quality of life [9,10,18]. In our previous research on HESA-A focusing on its antiviral activity against influenza A virus H1N1, the EC_50_ (effective concentration) of this compound was obtained at 0.025 mg/ml with no cytotoxic effect on MDCK cells and its anti-inflammatory effects against two important inflammatory cytokines; TNF-α and IL-6 was verified in genome and protein level in 1 hr exposure time. The results showed the inhibitory effects of HESA-A on H1N1 infection. In the present study we confirmed the inhibitory effects of HESA-A on influenza infection by different types of combination treatments in different time points. Data from viability test showed that co- & pre-treatments but not post-treatment, even in short incubation times protected the cell viability against H1N1 CPE significantly (p ≤ 0.05). However, data from HA assay showed highly significant decrements in HA titers in all combination treatments even post-treatment (p ≤ 0.01). From these results it is postulated that post-treatment cannot protect the cell viability from viral CPE, therefore, the decrease in HA titer in this type of exposure means no viable cell presence to support the viral replication. In conclusion, supporting achievements from previous study which showed the effects of HESA much stronger than amantadine and also supporting data from current study, suggest that HESA-A as a natural product has the potential of supplementary medication or alternative to antiviral agents to inhibit influenza infection especially in the first steps of its infection cycle by prophylactic effects affecting the virus penetration/adsorption. More detailed investigation on viral and cellular pathways could be of interest to evaluate the localization of function of HESA-A on virus-host interaction system.

## Conclusion

In conclusion, supporting achievements from previous study which showed the effects of HESA much stronger than amantadine and also supporting data from current study, suggest that HESA-A as a natural product has the potential of supplementary medication or alternative to antiviral agents to inhibit influenza infection especially in the first steps of its infection cycle by prophylactic effects affecting the virus penetration/adsorption. More detailed investigation on viral and cellular pathways could be of interest to evaluate the localization of function of HESA-A on virus-host interaction system.

## Abbreviations

ANOVA: Analysis of variance; ATCC: American Type Culture Collection; CPE: Cytopathic effect; DMEM: Dulbecco's modified Eagle’s medium; EC50: Effective concentration; FBS: Fetal bovine serum; HA: Hemagglutination assay; MDCK: Madin-Darby Canine Kidney; TPCK: Tosylamide Phenylethyl Chloromethyl Keton-treated Trypsin.

## Competing interests

The authors declare that they have no competing interests.

## Authors’ contributions

PM and AI co-defined the research theme. PM designed the methods and experiments. She carried out the laboratory experiments, worked on the associated data collection and their interpretation and drafted the manuscript. PM, AI, ARO and MH-B revised the paper critically for important intellectual content. All authors have seen and approved the manuscript.

## Pre-publication history

The pre-publication history for this paper can be accessed here:

http://www.biomedcentral.com/1472-6882/14/131/prepub
